# Rice protein concentrate is a well-accepted, highly digestible protein source for adult cats

**DOI:** 10.3389/fvets.2023.1168659

**Published:** 2023-04-28

**Authors:** Elizabeth Morris, Sunil Perumalla, Cheryl Stiers, Kathy Gross

**Affiliations:** ^1^Hill’s Pet Nutrition, Inc., Topeka, KS, United States; ^2^Department of Animal Science & Industry Adjunct Faculty, Kansas State University, Manhattan, KS, United States

**Keywords:** macronutrient digestibility, rice protein concentrate, novel ingredient, feline, cat

## Abstract

**Introduction:**

The use of rice protein concentrate (RPC) as a protein source in cat food is uncommon. Therefore, this study aimed to determine the acceptability and digestibility of foods formulated to contain increasing levels of RPC to support its inclusion in foods for adult (non-gravid, non-lactating) cats.

**Methods:**

Increasing levels of RPC (0, 7, 14, and 28%) were formulated into test foods fed to 24 cats in a Latin square design with 15-day periods and no washout between periods. Food intake and fecal scores were measured to determine the acceptability of test foods. Fecal output was measured on days 11–15. Food and fecal samples from day 15 of each period were analyzed for nutrient composition to calculate the macronutrient digestibility of the test foods. Analysis of variance and orthogonal contrasts were used to assess the effects of RPC inclusion on food intake, fecal output, fecal scores, and macronutrient digestibility.

**Results:**

The results showed that as-fed (AF), dry matter (DM), and gross energy (GE) intake increased with increasing RPC levels (*p* > 0.05). Fecal output, both as-is and DM, was unaffected by RPC inclusion (*p* > 0.05); however, fecal scores increased linearly with increasing RPC inclusion (*p* < 0.001). Furthermore, true protein and apparent DM, GE, and carbohydrate (NFE) digestibility increased linearly with RPC inclusion (*p* < 0.05). Apparent fat digestibility was high for all test foods but was unaffected by RPC inclusion (*p* = 0.690).

**Discussion:**

Overall, the inclusion of RPC was well-accepted, improved fecal characteristics, and increased the apparent and true macronutrient digestibility compared to the control. Therefore, this study demonstrated that RPC can serve as a high-quality and acceptable protein source for adult cats.

## 1. Introduction

A consistent supply of good quality protein with uniform amino acid content is required to formulate high-quality foods designed to meet the nutritional needs of cats. To this end, sources of plant proteins, such as corn gluten meal, wheat gluten, pea protein, and potato protein, are already widely used and accepted in commercial cat foods (1). The inclusion of plant proteins in food can reduce the requirement for animal protein sources and may have a less negative impact on the environment (2).

From a clinical standpoint, cats with food intolerances, gastritis, enteritis, or other chronic conditions need a source of protein that is palatable, maximally digestible, and that they can tolerate (3). As a result, therapeutic foods for these indications often utilize alternative or hydrolyzed protein sources to reduce the likelihood of adverse food reactions (4–6). Since the digestibility of plant proteins has been observed to be comparable to that of animal proteins in feline foods (1), it is worth investigating the inclusion of concentrated plant proteins such as rice protein concentrate (RPC) in feline foods formulated for these conditions.

Rice protein concentrate is sourced from rice or rice brokens, which are co-products of milling husked rice to produce white rice. To prepare RPC, rice is first soaked in water and then ground; protein is then solubilized and insoluble starch is decanted. The protein is then recovered by precipitation and dried to form a concentrated protein powder (7). The resulting RPC is a high-quality protein source that provides an amino acid (AA) content comparable to that of other commonly used protein sources in pet foods (7, 8). Hydrolyzed rice protein and rice protein concentrate have been investigated and are currently used as alternatives to cow’s milk or soy protein in infant formulas. They are considered to be safe, viable alternatives for infants with cow’s milk protein allergy (9–11). Rice protein concentrate has also been evaluated as a protein source in the diets of weaned pigs and farmed fish, like rainbow trout and sea bass. These investigations report that RPC could be used as a replacement for other protein sources, such as dried whey and fish meal, without adverse effects on health or growth performance (12–16). These studies also highlighted an important limitation of RPC as a protein source, which is that it may be lacking in some essential amino acids, such as lysine, thus emphasizing the importance of including other protein or amino acid sources as needed to ensure that amino acid requirements are met.

However, information regarding the use of RPC in feline foods is limited. Although the digestibility of cat and dog foods containing RPC and other plant-based proteins has been investigated (1), the effects of increasing RPC levels on acceptability and digestibility in cats are unknown. This study aimed to determine the acceptability, metabolizable energy, and macronutrient digestibility of feline foods containing increasing levels of RPC. The digestibility of macronutrients in the RPC ingredient itself was estimated using regression analyses. It was hypothesized that the inclusion of RPC would not negatively affect the acceptability or macronutrient digestibility of the test foods.

## 2. Materials and methods

### 2.1. Humane consideration

This study was conducted with the approval of Hill’s Pet Nutrition Institutional Animal Care and Use Committee (IACUC) and in accordance with Hill’s Global Animal Welfare Policy. At no time was any animal subjected to procedures expected to cause pain or distress.

### 2.2. Animals

Cats of at least 1 year of age, fully grown, in good health, and with a known weight were considered for inclusion in this study. Cats were excluded from the study if they had been diagnosed with a chronic disease, including, but not limited to, kidney disease, cancer, hyperthyroidism, and diabetes. Cats enrolled in the study were subsequently removed from the study if they: (1) experienced excessive weight loss (>1.5% per week); (2) stopped eating for 2 days or ate less than 50% of the food offered for 3 days; or (3) were subsequently diagnosed with any secondary systemic disease as described in the ‘exclusion criteria’ mentioned above. All cats were immunized against Rabies (rabies), Felid alphaherpesvirus 1 (viral rhinotracheitis), Feline caliciviridae (feline calicivirus), and Feline panleukopenia (feline panleukopena virus). None of the cats had chronic systemic disease, as evaluated by annual urinalysis, serum biochemical analyses, complete blood count, and physical examination. The cats were housed individually, owned and maintained by Hill’s Global Pet Nutrition Center, and treated in accordance with Hill’s Global Animal Welfare Policy. While individually housed, the cats had access to group socialization and interaction with animal care technicians and toys. The study design did not interfere with the daily routines of the animals. The cats were housed in temperature-controlled facilities with access to natural light.

Twenty-six domestic shorthair cats (15 spayed females, 11 neutered males; 5.1 ± 0.9 kg body weight; 7.7 ± 2.2 years of age) were initially enrolled in this study, and a summary of their signalment is shown in Table 1. Two cats were removed from the study: one was removed because of a health concern that required an antibiotic regimen (deemed by the attending veterinarian to be unrelated to the test food), and the cat selected to replace the first cat was subsequently removed from the study after poor eating as outlined above. Thus, a total of 24 cats completed the study and were included in the statistical analysis. Following enrollment, the cats were fed once daily to maintain their ideal body weight based on the daily metabolizable energy requirements of adult cats at maintenance, calculated as (70 × BW^0.75^) × 1.2, and the food intake was manually recorded each day by an animal care technician. Water was provided *ad libitum*.

**Table 1:** Signalment for each panel of cats.

Signalment	Overall	By panel			
		1	2	3	4
Animals	26	6	6	6	8^1^
Male	11	3	2	4	2
Female	15	3	4	2	6
Body weight, kg	5.1 ± 0.9	5.5 ± 0.8	4.7 ± 0.4	5.1 ± 0.6	5.0 ± 1.3
Average age, yr	7.7 ± 2.2	7.5 ± 1.0	8.8 ± 3.2	8.0 ± 2.4	6.8 ± 1.3

1 Includes two cats removed from the study whose data were not included in the statistical analysis.

Data are represented as absolute counts or as mean ± standard deviation.

### 2.3. Study design

This study used the American Association of Feed Control Officials (AAFCO) quantitative collection protocol (17) with a minimum of six adult cats. This protocol consists of two phases. The pre-collection phase lasts for at least 5 days and allows the cats to become acclimated to the test food. Food intake is then adjusted as needed to ensure that weight is neither gained nor lost. The next five-day phase is used for total fecal collection. Food offerings remain unchanged during the fecal collection phase (based on the amount needed to maintain weight from the earlier phase).

In this study, 24 adult male and female cats were grouped into four panels (*n* = 6 cats per panel) that were arranged in a 4 × 4 Latin square design. Each panel was fed each test food for 15 days with no washout between periods. Body weight (BW) was measured on days 1, 8, and 15 during each period. Fecal scores were recorded on days 10–15 using a 6-point scale (1 = liquid diarrhea, 2 = soft feces lacking form, 3 = soft, moist, but formed feces, 4 = firm, well-formed feces, 5 = hard, segmented feces, and 6 = constipation with no feces). Fecal scores of 3–5 were considered normal. Feces were collected from each cat individually using non-absorbent beads (Providence House Manufacturing, Inc., Seal Rock, OR, USA) in a litterbox and were weighed on days 11–15. Cats were individually fed once daily with access to food for approximately 22 h each day, and their food intake was recorded. Food and fecal samples from day 15 were analyzed for nutrient composition and subsequent calculation of digestibility.

### 2.4. Study foods

The study foods were manufactured using a Wenger X-115 extruder (Wenger Manufacturing, Inc.; Sabetha, KS, USA) according to the manufacturing procedures outlined in Supplementary Table 1. Test foods were formulated to meet or exceed AAFCO requirements for adult cats at maintenance, with the major ingredients and nutrient profiles of the study foods are summarized in Tables 2, 3, respectively. The analyzed nutritional composition of the RPC included in the test foods on a dry matter basis (DMB) is provided in Supplementary Table 2 and compared to grade A whole large eggs (18) as an indicator of protein quality.

**Table 2:** Select ingredients included in test foods containing increasing levels of rice protein concentrate.

	Rice protein concentrate inclusion			
Ingredient, %	0%	7%	14%	28%
Hydrolyzed chicken liver and heart	49.4	39.0	27.1	6.1
Rice, brewers	31.0	34.1	38.4	45.3
Rice protein concentrate	0.0	7.0	14.0	28.0
Taurine	0.2	0.2	0.2	0.2
Soybean oil	3.8	3.8	3.8	3.8
Coconut oil	2.4	2.4	2.4	2.4
Vitamin premixes^1^	0.4	0.4	0.5	0.4
Mineral premixes^2^	0.3	1.2	1.8	1.9

1 Vitamin premixes included rice hulls as a carrier for this blend. The individual vitamin compounds included vitamin A, vitamin D, vitamin E, thiamine, riboflavin, pyridoxine, vitamin B12, ascorbic acid, niacin, pantothenic acid, folic acid, biotin, and beta-carotene.

2 Mineral premixes included calcium carbonate as a carrier for this blend. The individual mineral compounds included ferrous sulfate, zinc oxide, copper sulfate, manganese oxide, calcium iodate, and sodium selenite.

Values are shown as percent of the total recipe. All test foods were formulated to meet or exceed AAFCO requirements for adult cats at maintenance (17).

**Table 3:** Analyzed nutrient composition and energy content of test foods containing increasing levels of rice protein concentrate (RPC) on a dry matter basis.

	AAFCO minimum^1^		Rice protein concentrate inclusion			
Nutrient		Unit	0%	7%	14%	28%
DM		%	90.2	92.3	92.1	93.3
Calories		kcal/kg	4,542	4,960	4,828	4,718
Protein (crude)	26.0	%	32.1	33.4	30.8	33.7
Fat (crude)	9.0	%	19.5	19.0	16.0	12.2
Fiber (crude)		%	2.1	2.7	2.4	3.0
Total dietary fiber		%	5.2	5.3	5.2	5.2
Methionine + cysteine	0.40	%	1.0	1.3	1.2	1.4
Phenylalanine + tyrosine	1.53	%	2.5	2.7	2.6	3.0
Arginine	1.04	%	1.8	2.2	2.1	2.5
Histidine	0.31	%	0.7	0.8	0.7	0.8
Isoleucine	0.52	%	1.3	1.5	1.3	1.2
Leucine	1.24	%	2.8	2.8	2.5	2.6
Lysine	0.83	%	2.2	2.1	1.7	1.4
Threonine	0.73	%	1.4	1.4	1.3	1.2
Tryptophan	0.16	%	0.4	0.5	0.5	0.5
Valine	0.62	%	1.7	1.9	1.7	1.8
Taurine	1,000	ppm	4,000	4,000	3,200	2,400
Calcium	0.6	%	0.8	0.8	0.8	0.8
Phosphorous	0.5	%	0.8	0.8	0.8	0.7
Potassium	0.6	%	0.9	1.0	0.9	0.8
Sodium	0.2	%	0.4	0.4	0.4	0.3

1 All test foods were formulated to meet or exceed AAFCO requirements for adult cats at maintenance (17).

### 2.5. Sample analysis

Measurements of ash, crude fiber, fat, protein, moisture, dry matter, and gross energy were completed for both food and fecal samples by a commercial laboratory (Eurofins Scientific, Inc., Des Moines, IA, USA) using official methods of analysis published by AOAC International (19).

The apparent digestibility of dry matter (DM), fat, gross energy (GE), and carbohydrates (nitrogen-free extract or NFE) was calculated as follows:


(1) Apparent digestibility(%)=[(intake−fecal output)/intake]×100

To correct for endogenous metabolic fecal protein (i.e., fecal protein of non-dietary origin), an endogenous protein correction of 63 mg nitrogen/kg weight to the ¾ power suggested by Kendall et al. was used (20). This value is in the range of estimates for fecal metabolic protein for both the dog and cat (21, 22). Therefore, true protein digestibility was calculated by subtracting an estimate of the metabolic protein contained in the feces from the measured fecal protein concentration. This was calculated as follows:


(2) $$\begin{array}{l} \text{True protein digestibility}\;(\%)=\\ \left\{\begin{array}{l} \left[\text{protein intake}-(\text{fecal protein}-\text{endogenous metabolic protein})\right]/\\ \text{protein intake} \end{array}\right\}\times 100 \end{array}$$

### 2.6. Statistical analysis

An investigation revealed an error in the fat analysis of the 28% RPC food sample collected during the first period of the study (3.1% crude fat compared to the mean of 12.2% crude fat from the three samples of the 28% RPC food collected during the other three periods of the study). This resulted in an artificial reduction in the subsequent calculation of apparent fat digestibility for that period. The error could not be rectified because the sample had already been destroyed. Therefore, data regarding fecal fat content from the first period for the panel consuming 28% RPC were removed from the analysis and were not included in the apparent fat digestibility calculations, as they were >3 standard deviations from the mean. All remaining data were analyzed using analysis of variance (ANOVA) of a linear model in R (v. 4.0.3) (23), including the fixed effects of diet, period, panel, and all associated interactions. The fixed effect panel was not significantly different (*p* > 0.05) and was removed from the model along with all associated interactions. Orthogonal contrasts were used to determine the linear, quadratic, or cubic relationships. The macronutrient digestibility of 100% RPC was predicted using linear regression analysis. The effects were considered significant when *p* ≤ 0.05. The results were presented as mean ± standard deviation or standard error, as appropriate.

## 3. Results

### 3.1. Food intake and fecal characteristics

The mean food intake and fecal characteristics are presented in Table 4. Neither the period nor the interaction of treatment by period affected body weight, food intake or fecal characteristics (*p* > 0.05). Mean food offered (63.4 ± 5.1 g as-fed/d) and BW (5.1 ± 0.3 kg) were similar between treatments (*p* = 0.278 and 0.365, respectively). As-fed (AF), DM, and GE intakes increased with RPC inclusion compared to control (*p* = 0.040, 0.017, and 0.040, respectively) but were similar between RPC inclusion levels (*p* = 0.880, 0.957, and 0.879, respectively). Fecal output, both as-is and DM, was unaffected by the inclusion of RPC (*p* = 0.267 and 0.685, respectively). With increasing RPC inclusion, fecal moisture decreased linearly (*p* = 0.001), whereas fecal scores increased linearly (*p* < 0.001). However, all mean fecal scores were considered normal for all treatments.

**Table 4:** Mean intake, fecal output, and fecal scores of cats consuming increasing levels of rice protein concentrate.

	Rice protein concentrate					*p*-Value					
Item	0%	7%	14%	28%	SE	Treatment	Linear	Quadratic	Cubic	Period	Treatment × period
Body weight, kg	5.1	5.1	5.1	5.1	0.27	0.365	0.053	0.108	0.371	0.781	0.808
Intake											
Intake, g AF/d^1^	54.0^a^	58.8^b^	60.7^b^	61.1^b^	1.01	0.040	0.880	0.104	0.974	0.618	0.129
Intake, g DM/d	49.2^a^	54.7^b^	55.9^b^	57.2^b^	1.04	0.017	0.957	0.082	0.717	0.118	0.124
GE Intake, kcal/d	270.3^a^	294.0^b^	302.1^b^	305.6^b^	5.05	0.040	0.879	0.104	0.974	0.142	0.129
Output											
Fecal output, g as-is/d	40.6	39.4	36.2	34.5	1.28	0.267	0.171	0.743	0.816	0.267	0.103
Fecal output, g DM/d	13.9	14.2	13.1	13.1	0.40	0.685	0.540	0.819	0.518	0.316	0.809
Fecal moisture, %	65.7^a^	63.8^b^	63.7^b^	61.9^c^	0.40	0.001	0.002	0.852	0.534	0.402	0.811
Fecal score	3.9^a^	4.2^b^	4.3^b,c^	4.6^c^	0.08	<0.001	<0.001	0.274	0.087	0.720	0.088

1 AF = as fed; DM = dry matter; GE = gross energy.

^a-c^Means within a row with different letters indicate a difference at *p* ≤ 0.05.

Fecal scores were determined using a 6-point scale (1 = liquid diarrhea, 2 = soft feces lacking form, 3 = soft, moist, but formed feces, 4 = firm, well-formed feces, 5 = hard, segmented feces, and 6 = constipation with no feces). Fecal scores of 3–5 were considered normal.

### 3.2. Macronutrient digestibility

The apparent macronutrient digestibility and true protein digestibility of test foods with increasing levels of RPC are presented in Table 5. Neither the period nor the interaction of treatment by period affected digestibility of any macronutrients (*p* > 0.05). Except for apparent fat digestibility (*p* = 0.690), the digestibility of all macronutrients increased linearly with increasing RPC inclusion (*p* < 0.05).

**Table 5:** Total tract apparent and true macronutrient digestibility in cats consuming increasing levels of rice protein concentrate.

	Rice protein concentrate					*p*-Value					
Digestibility, %	0%	7%	14%	28%	SE	Treatment	Linear	Quadratic	Cubic	Period	Treatment × Period
Apparent DM	80.1^a^	82.7^b^	84.3^b^	85.1^b^	0.62	0.008	0.029	0.478	0.845	0.678	0.465
True protein	90.3^a^	92.4^a,b^	93.7^b^	95.8^c^	0.61	0.003	0.018	0.815	0.536	0.676	0.462
Apparent fat	91.0	91.0	91.7	89.8	0.34	0.289	0.690	0.193	0.231	0.228	0.989
Apparent carbohydrate (NFE)	83.5^a^	89.0^b^	90.5^b,c^	92.8^c^	0.97	< 0.001	<0.001	0.131	0.135	0.834	0.256
Apparent GE	83.6^a^	86.0^b^	87.0^b^	87.8^b^	0.51	0.008	0.038	0.364	0.622	0.625	0.571

^a-c^Means within a row with different letters indicate a difference at *p* ≤ 0.05.

### 3.3. Predictions of digestibility of RPC

The test food recipes were designed with increasing levels of RPC (0, 7, 14, and 28%), with adjustments to the amount of hydrolyzed chicken liver and heart, and rice in the recipe to create test foods with similar levels of total protein. This allowed a prediction to be made regarding the apparent macronutrient digestibility of 100% RPC (Table 6). The predicted value in adult cats was high, with predicted dry matter digestibility above 85%, predicted fat digestibility above 90%, and predicted protein and NFE digestibility above 95%. Since there was no effect of RPC on apparent fat digestibility, the *R*^2^ for predicted apparent fat digestibility was low (*R*^2^ = 0.07), indicating it is a poor model for estimating the apparent fat digestibility of 100% RPC.

**Table 6:** Prediction of apparent macronutrient digestibility of 100% rice protein concentrate.

Nutrient	Linear equation	SE	Adjusted *R*^2^	Predicted digestibility, %
Dry matter	*Y* = 83.03 + 0.038*x*	1.61	0.57	86.8
True protein	*Y* = 93.01 + 0.040*x*	1.44	0.65	97.0
Fat	*Y* = 90.89–0.0066*x*	1.25	0.07	90.2
Carbohydrate	*Y* = 88.94 + 0.065*x*	1.84	0.78	95.5

## 4. Discussion

### 4.1. Characteristics of RPC

Rice protein concentrate can be considered a high-quality protein source because it is an excellent source of essential AAs. Compared to Grade A large whole eggs, which may be considered the gold standard in protein quality and digestibility for both human and pet nutrition (8, 24), the nutritional composition of the RPC used in this study meets or exceeds the DM AA content of whole eggs in all but two AAs: lysine and cysteine (10). It is common for plant-based proteins to be deficient in at least one essential amino acid (8, 25), further emphasizing the importance of ensuring that foods utilizing plant proteins such as RPC are formulated to be complete and balanced, which has been recommended when other plant protein sources have been investigated for use in companion animal foods (25). For example, all four test foods in this study were supplemented with taurine at the same level (0.2% of the recipe), but inclusion of brewers rice and RPC in test foods (at the expense of hydrolyzed chicken liver and heart) reduced the taurine content of the 14 and 28% foods as neither brewers rice nor RPC contains no taurine. However, all AA levels in these test foods, including taurine, exceeded the minimum recommended allowance of the National Research Council (26) and the AAFCO minimum (17) for adult cats at maintenance as a result of the supplemental taurine added to the test foods.

### 4.2. Food intake and fecal characteristics

Overall, the study foods were well-accepted by all cats. While the amount of food offered was similar between treatments, AF and DM intakes increased with RPC inclusion compared with those in the control food, which resulted in greater gross energy intake when cats were fed RPC-inclusive foods. Since cats were fed to maintain their ideal body weight, this may indicate a preference for foods with higher levels of RPC, which may be of particular interest because cats are often picky eaters (27). However, the increased energy intake did not translate into an increase in BW. This was most likely a result of the short experimental period (15 d). A similar effect was reported by Detweiler et al. (28), who reported lower food intake but no change in BW when cats were fed an experimental food formulated with beet pulp as the main source of dietary fiber. One notable limitation of the current study is that neither body condition score nor muscle condition score were assessed. It would be prudent in future work to include such measures in addition to BW in order to assess the potential of RPC to affect lean body mass or fat mass. The increase in intake also did not translate into an effect on fecal output (for as-is or DM). These data were consistent with the increase in DM digestibility with increasing RPC inclusion in the test foods. The fecal scores, while considered normal (stool scores of 3–5 on a 1–6 scale) for all test foods throughout the study, increased linearly with increasing RPC inclusion, which is consistent with the corresponding decrease in fecal moisture. The increased fecal score was likely due to high digestibility, reduced hydrolysate content, and slightly higher crude fiber content in the recipes containing RPC. While an increase in dietary fiber is associated with increased fecal output (29–31), it is likely that the fiber differences in these test foods were not substantial enough to impact as-is fecal output as seen in these other studies.

### 4.3. Macronutrient digestibility

All study foods were highly digestible, with the digestibility of all macronutrients above 80%. Except for apparent fat, the digestibility of all macronutrients increased linearly with increasing RPC inclusion. Apparent fat digestibility was high (>90%) in all test foods and was unaffected by RPC inclusion level, though this may be a result of the error in food sample analysis that resulted in the removal of one 28% RPC period’s apparent fat digestibility data. This can be avoided in future work through the closer inspection of results and retention of samples to re-analyze if necessary. While the predictive equation for apparent fat should not be used due to the absence of an effect of RPC on apparent fat digestibility as well as the poor fit of the linear regression predictive equation for fat digestibility, the predicted macronutrient digestibility of 100% RPC was high for all other macronutrients (above 85% for DM and above 95% for both protein and carbohydrate). The hydrolyzed chicken ingredient was specifically chosen as the other primary protein source because hydrolyzed meat ingredients are considered highly digestible protein sources and are commonly used in foods for cats with food sensitivities as proteins with lower molecular weight (< 10,000 daltons) are less likely to elicit an immune response (32). Thus, it is interesting that replacing the hydrolyzed chicken ingredient with RPC resulted in higher protein digestibility. However, all test foods, including the control that had the hydrolyzed chicken ingredient as the primary protein source, demonstrated true protein digestibility above 90%. These results do not indicate that hydrolyzed meat ingredients are poorly digestible, but rather that molecular weight of an ingredient is not the only factor determining the digestibility of a protein source. These data are consistent with previous investigations on the digestibility of plant and animal proteins in feline foods, in which protein digestibility in cats increased with the percentage of dietary protein from plant-based sources (1). As with AA content of the ingredient, RPC may also be comparable to whole eggs in terms of protein digestibility. Previous work has shown that when used in dog food at 20% of total crude protein, an egg-inclusive food had a total tract apparent protein digestibility of 91.2% (33).

In conclusion, adult cats were fed foods containing increasing levels of RPC up to 28% of the total recipe, and it was demonstrated that RPC could serve as a protein source in cat foods owing to its high digestibility and protein quality. Overall, the inclusion of RPC was well-accepted by cats, improved fecal characteristics, and increased the apparent and true macronutrient digestibility compared with the control food that did not contain RPC. Thus, RPC may serve as a complementary protein source in feline foods considering its AA content, good digestibility, and excellent taste acceptance, all of which make it appropriate for use in therapeutic foods indicated for cats with food sensitivities. Future directions include the investigation on the effect of RPC inclusion on lean body mass in cats.

## Data availability statement

The raw data supporting the conclusions of this article will be made available by the authors, without undue reservation.

## Ethics statement

The animal study was reviewed and approved by Hill’s Pet Nutrition Institutional Animal Care and Use Committee.

## Author contributions

KG and SP: conceptualization. KG, SP, and CS: methodology, writing—review and editing. EM: formal analysis, project administration, and writing—original draft preparation. KG and CS: investigation. EM and KG: data curation. KG: supervision. All authors contributed to the article and approved the submitted version.

## Funding

The study was funded by Hill’s Pet Nutrition, Inc. The funder employed or funded the active participation of all authors in the study and in the development of the manuscript.

## Conflict of interest

EM, SP, and CS are employed by Hill’s Pet Nutrition, Inc. KG is a former employee of Hill’s Pet Nutrition, Inc. The authors declare that this study received funding from Hill’s Pet Nutrition, Inc. The funder was provided an opportunity to review the manuscript for appropriate animal welfare documentation but was not involved in the study design, collection, analysis, interpretation of data, the writing of this article, or the decision to submit it for publication.

## Publisher’s note

All claims expressed in this article are solely those of the authors and do not necessarily represent those of their affiliated organizations, or those of the publisher, the editors and the reviewers. Any product that may be evaluated in this article, or claim that may be made by its manufacturer, is not guaranteed or endorsed by the publisher.
