# Supplementary material for: Rice protein concentrate is a well-accepted, highly digestible protein source for adult cats
Source: Front Vet Sci. 2023 Apr 28;10:1168659. doi: 10.3389/fvets.2023.1168659 (PMC10175793; doi:10.3389/fvets.2023.1168659)
Supplement: Supplementary file 1 [file Table_1.docx]

Supplementary Material

Rice protein concentrate is a well-accepted, highly digestible protein source for adult cats

Elizabeth Morris*, Sunil Perumalla, Cheryl Stiers, and Kathy Gross

*** Correspondence:** Elizabeth Morris: [elizabeth_morris@hillspet.com](mailto:elizabeth_morris@hillspet.com)

# Supplementary tables

**Supplementary Table 1**. Production process specifications for the test foods produced using a Wenger X-115 (Wenger Manufacturing, Inc.; Sabetha, KS, USA).

| **Die Assembly** | Number of Knives | 6 |
| --- | --- | --- |
|  | New Die Hole Altitude (inch): | 0.272 |
|  | Used Die Holes Size (maximum): | 4 |
|  | Breaker plate | yes |
| **Preconditioner & Extruder** |  | **TARGET** |
|  | Dry Mix Feed Rate (kg/min): | 32 |
|  | Preconditioner Steam (%): | 7 |
|  | Preconditioner Water (%): | 0 |
|  | Preconditioner Exit Throat Temp (F): | 190 |
|  | Extruder Steam (%): | 0 |
|  | Extruder Water (%): | 0 |
|  | Extruder RPM (Fixed): | 270 |
|  | Extruder Load (%) SME Level (BTU/#): | 28% |
|  | BPV Setting: | 100% |
|  | Extruder Temp Set Point (F) -  Die Temp | 245 |
|  | Head #4: | 200 |
|  | Head #5: | 245 |
|  | Head #6: | 265 |
|  | Head #7: | 230 |
|  | Wet density EFL Method - (lb/cuft): | 26.8 |
| **Drying** | Dryer Type: | 3 pass |
|  | Temperature Setting - Zone #1 (F): | 121 |
|  | Zone #2 (F): | 132 |
|  | Zone #3 (F): | 125 |
|  | Dryer Time | Dampers in the 60 40 25 5 positions |
|  | Belt Speed - Top Belt (ft/min): | 3:55 |
|  | Middle Belt (ft/min): | 3:55 |
|  | Bottom Belt (ft/min): | 3:55 |
