# Supplementary material for: Rice protein concentrate is a well-accepted, highly digestible protein source for adult cats
Source: Front Vet Sci. 2023 Apr 28;10:1168659. doi: 10.3389/fvets.2023.1168659 (PMC10175793; doi:10.3389/fvets.2023.1168659)
Supplement: Supplementary file 2 [file Table_2.docx]

Supplementary Material

Rice protein concentrate is a well-accepted, highly digestible protein source for adult cats

Elizabeth Morris*, Sunil Perumalla, Cheryl Stiers, and Kathy Gross

*** Correspondence:** Elizabeth Morris: [elizabeth_morris@hillspet.com](mailto:elizabeth_morris@hillspet.com)

# Supplementary tables

**Supplementary Table 2.** Nutrient composition of rice protein concentrate included in test foods on a dry matter basis (DMB). For comparison, the composition of a Grade A, whole, large egg is included on a DMB (USDA, 2019).

| **Nutrient** | **Unit** | **Rice Protein Concentrate** | **Egg** |
| --- | --- | --- | --- |
| Energy (Atwater) | kcal/kg | 3423 | 6074 |
| Protein (Crude) | % | 89.9 | 51.2 |
| Fat (Crude) | % | 8.1 | 41.2 |
| Fiber (Crude) | % | 1.2 | 0.0 |
| Ash | % | 1.3 | 3.5 |
| Methionine + Cystine | % | 3.5 | 3.3 |
| Phenylalanine + Tyrosine | % | 8.8 | 4.8 |
| Arginine | % | 6.9 | 3.3 |
| Histidine | % | 1.9 | 1.2 |
| Isoleucine | % | 3.4 | 2.5 |
| Leucine | % | 6.9 | 4.3 |
| Lysine | % | 2.9 | 3.4 |
| Methionine | % | 2.0 | 1.7 |
| Phenylalanine | % | 4.5 | 2.7 |
| Threonine | % | 3.0 | 2.5 |
| Tryptophan | % | 1.0 | 0.7 |
| Valine | % | 4.9 | 3.0 |
| Alanine | % | 4.6 | 2.8 |
| Aspartate | % | 7.6 | 5.2 |
| Cystine | % | 1.5 | 1.6 |
| Glutamine + Glutamate | % | 14.7 | 6.7 |
| Glycine | % | 3.6 | 1.7 |
| Proline | % | 3.7 | 2.3 |
| Serine | % | 4.2 | 3.8 |
| Tyrosine | % | 4.2 | 2.1 |
